# Device-Measured Sleep Characteristics, Daily Step Count, and Cardiometabolic Health Markers: Findings From the Prospective Physical Activity, Sitting, and Sleep (ProPASS) Consortium

**DOI:** 10.1161/CIRCOUTCOMES.124.011873

**Published:** 2025-07-24

**Authors:** Wenxin Bian, Matthew N. Ahmadi, Raaj Kishore Biswas, Joanna M. Blodgett, Andrew J. Atkin, Hsiu-Wen Chan, Borja del Pozo Cruz, Kristin Suorsa, Esmée A. Bakker, Richard M. Pulsford, Gregore I. Mielke, Peter J. Johansson, Pasan Hettiarachchi, Nicholas A. Koemel, Dick H.J. Thijssen, Sari Stenholm, Gita D. Mishra, Armando Teixeira-Pinto, Vegar Rangul, Lauren B. Sherar, Ulf Ekelund, Alun D. Hughes, I-Min Lee, Peter A. Cistulli, Andreas Holtermann, Annemarie Koster, Mark Hamer, Emmanuel Stamatakis, on behalf of the ProPASS Collaboration

**Affiliations:** 1 Mackenzie Wearables Research Hub, Charles Perkins Centre (W.B., M.N.A., R.K.B., N.A.K., E.S.), The University of Sydney, NSW, Australia.; 2 Faculty of Medicine and Health, School of Health Sciences (W.B., M.N.A., R.K.B., N.A.K., E.S.), The University of Sydney, NSW, Australia.; 3 Faculty of Medicine and Health, School of Public Health (A.T.-P.), The University of Sydney, NSW, Australia.; 4 Faculty of Medicine and Health, Northern Clinical School (P.A.C.), The University of Sydney, NSW, Australia.; 5 Sleep Research Group, Charles Perkins Centre (P.A.C.), The University of Sydney, NSW, Australia.; 6 Division of Surgery and Interventional Sciences, Institute of Sport Exercise and Health (J.M.B., M.H.), UCL, United Kingdom.; 7 Medical Research Council Unit for Lifelong Health and Ageing, University College London (UCL) Institute of Cardiovascular Science (A.D.H.), UCL, United Kingdom.; 8 UCL British Heart Foundation Research Accelerator (A.D.H.), UCL, United Kingdom.; 9 UCL Hospitals National Institute for Health and Care Research Biomedical Research Centre, United Kingdom (J.M.B., M.H., A.D.H.).; 10 School of Health Sciences, University of East Anglia, Norwich, United Kingdom (A.J.A., G.I.M.).; 11 School of Public Health, The University of Queensland, Brisbane, Australia (H.-W.C., G.D.M.).; 12 Faculty of Medicine, Health, and Sports, Department of Sport Sciences, Villaviciosa de Odón, Madrid, Spain (B.d.P.C.).; 13 Department of Public Health (K.S., S.S.), University of Turku and Turku University Hospital, Finland.; 14 Centre for Population Health Research (K.S., S.S.), University of Turku and Turku University Hospital, Finland.; 15 Department of Primary and Community Care (E.A.B.), Radboud University Medical Center, Nijmegen, the Netherlands.; 16 Department of Medical BioSciences, Exercise Physiology Research Group (D.H.J.T.), Radboud University Medical Center, Nijmegen, the Netherlands.; 17 Faculty of Sport Sciences, Department of Physical Education and Sports, Sport and Health University Research Institute, University of Granada, Spain (E.A.B.).; 18 Faculty of Health and Life Sciences, University of Exeter, United Kingdom (R.M.P.).; 19 Occupational and Environmental Medicine, Department of Medical Sciences, Uppsala University, Sweden (P.J.J., P.H.).; 20 Occupational and Environmental Medicine, Uppsala University Hospital, Sweden (P.J.J.).; 21 Faculty of Medicine and Health Sciences, Department of Public Health and Nursing, Trøndelag Health Study Research Centre, Norwegian University of Science and Technology, Levanger (V.R.).; 22 School of Sport, Exercise and Health Sciences, Loughborough University, United Kingdom (L.B.S.).; 23 NIHR Leicester Biomedical Research Centre, University Hospitals of Leicester National Health Service Trust and the University of Leicester, United Kingdom (L.B.S.).; 24 Department of Sport Medicine, Norwegian School of Sport Sciences, Oslo (U.E.).; 25 Department of Chronic Diseases, Norwegian Public Health Institute, Oslo (U.E.).; 26 Division of Preventive Medicine, Brigham and Women’s Hospital and Harvard Medical School, Boston, MA (I-M.L.).; 27 Department of Epidemiology, Harvard T.H. Chan School of Public Health, Boston, MA (I-M.L.).; 28 Department of Respiratory and Sleep Medicine, Royal North Shore Hospital, St Leonards, NSW, Australia (P.A.C.).; 29 National Research Centre for the Working Environment, Copenhagen, Denmark (A.H.).; 30 Department of Social Medicine, Care and Public Health Research Institute, Maastricht University, the Netherlands (A.K.).; Department of Human Movement Sciences, School for Nutrition and Translational Research in Metabolism, Maastricht University, Maastricht, the Netherlands; Department of Internal Medicine, Maastricht University Medical Centre, Cardiovascular Research Institute Maastricht, Maastricht University, Maastricht, the Netherlands; Department of Internal Medicine, Maastricht University Medical Centre, Cardiovascular Research Institute Maastricht, Maastricht University, Maastricht, the Netherlands; Department of Medical BioSciences, Radboud University Medical Center, Nijmegen, the Netherlands

**Keywords:** accelerometry, cardiometabolic risk factors, cardiovascular diseases, epidemiology, exercise, public health, sleep duration

## Abstract

**BACKGROUND:**

Sleep and physical activity (PA) are important lifestyle-related behaviors that impact cardiometabolic health. This study investigated the joint associations of daily step count and sleep patterns (regularity and duration) with cardiometabolic biomarkers in adults.

**METHODS:**

We conducted a cross-sectional study using pooled data from the Prospective PA, Sitting, and Sleep Consortium, comprising 6 cohorts across Europe and Australia with thigh-worn accelerometry data collected between 2011 and 2021. The sleep regularity index, a metric that quantifies day-to-day sleep consistency, sleep duration (h/d), and steps (per day), was derived from the accelerometer data and categorized based on tertiles and sleep duration guidelines. We used multivariate generalized linear models to examine joint associations of sleep patterns and total daily step count with individual cardiometabolic biomarkers, including body mass index, waist circumference, total cholesterol, HDL (high-density lipoprotein) cholesterol, triglycerides, HbA1c (glycated hemoglobin), and a composite cardiometabolic health score (mean of the 6 standardized biomarker *Z* scores).

**RESULTS:**

The sample included 11 903 adults with a mean±SD age of 54.7±9.5 years, 54.9% female, a sleep regularity index of 78.7±10.4, and 10 206.4±3442.2 daily steps. Lower PA (<8475 steps/d) combined with either lower sleep regularity (sleep regularity index <75.9) or short sleep duration (<7 h/d) was associated with the least favorable composite cardiometabolic health. The corresponding *Z* scores (95% CI) were 0.34 (0.30–0.38) and 0.26 (0.22–0.31) compared with those with optimal sleep (sleep regularity index >84.5 or 7–8 h/d) and high step count (>11 553 steps/d). The combination of low sleep regularity and low daily steps was associated with higher body mass index (2.92 [2.61–3.24] kg/m^2^), waist circumference (8.58 [7.78–9.38] cm), total cholesterol (0.15 [0.07–0.23] mmol/L), and lower HDL levels (0.17 [0.14–0.2] mmol/L), regardless of sleep duration. The combination of short sleep and low step count had the strongest unfavorable associations for body mass index (2.31 [1.98–2.65] kg/m^2^) and waist circumference (7.01 [6.15–7.87] cm).

**CONCLUSIONS:**

Our findings suggest that the potential deleterious associations of irregular or insufficient sleep with cardiometabolic health outcomes may be exaggerated by lower daily PA. Investigation of the prospective joint association of sleep patterns and PA with cardiometabolic health may be warranted.

**What Is Known**

- Public health guidelines highlight the importance of accessible daily physical behaviors in health promotion and chronic disease prevention.
- Sleep and physical activity are a pair of physical behaviors with a synergistic and bidirectional relationship, yet are largely studied in isolation.
- The majority of sleep evidence has heavily relied on questionnaire-based measures and single sleep parameters and has failed to capture sleep-wake patterns across multiple sleep dimensions.

**What The Study Adds**

- Irregular sleep and insufficient sleep combined with low daily step count were jointly associated with higher body mass index, waist circumference, total cholesterol, lower HDL (high-density lipoprotein) levels, and a more adverse composite cardiometabolic health score.
- Potential detrimental associations of irregular or insufficient sleep with cardiometabolic health outcomes may be exaggerated by lower levels of daily physical activity.

Cardiometabolic diseases constitute a cluster of common, preventable, noncommunicable chronic conditions and are some of the main contributors to deaths worldwide.^1,2^ Key indicators of cardiometabolic risk, such as body mass index (BMI) and waist circumference, along with cardiometabolic blood biomarkers such as HDL (high-density lipoprotein) cholesterol, total cholesterol, triglycerides, and HbA1c (glycated hemoglobin), are crucial for understanding and identifying individuals’ cardiometabolic health issues. These indicators can be used for risk screening and identifying groups or individuals for targeted interventions in cardiometabolic health.

Physical activity (PA) is an important physical behavior that reduces the risk of cardiometabolic diseases in people of all ages.^3,4^ Among the many forms of PA, stepping behavior is considered one of the most common and accessible indicators for PA promotion.^5^ Various metrics related to stepping have been linked to health outcomes, such as stepping rate, volume, and stepping intensity.^6,7^ However, daily total step count has gained popularity for setting PA behavior change goals due to its intuitive nature in targeting specific step counts each day.^5^ Increasing daily step count has shown beneficial associations with markers of cardiometabolic risk, such as BMI, HbA1c, HDL, and triglycerides.^5,8^ Recent studies in middle-aged adults have found an L-shaped dose-response association between step count and cardiovascular disease (CVD) risk markers,^8^ CVD morbidity, and mortality.^9,10^

Sleep is a multidimensional behavior that is also recognized as a crucial risk factor for cardiometabolic health.^11,12^ Sleep regularity and sleep duration are 2 important (health-related) dimensions of sleep. Sleep regularity^13^ reflects the day-to-day consistency in sleep/wake pattern and has shown beneficial associations with both cardiometabolic and overall health.^14–16^ Sleep duration, which has been studied more extensively, appears to have a U-shaped association with adverse cardiometabolic health outcomes, such as obesity, hypertension, type 2 diabetes, and CVD.^17^ To date, the majority of epidemiological studies have used self-reported sleep data to estimate sleep parameters,^18^ which limits accurate capture of sleep-wake patterns. Moreover, wearables may provide more accurate data on sleep duration, as overreporting is common in the middle-aged,^19^ and a discrepancy of around an hour less in sleep duration was found with accelerometry measurements compared with questionnaire reports.^20^

There is an evidence gap regarding the combined associations of sleep and PA with cardiometabolic markers, as they have primarily been examined independently. However, one behavior by itself cannot determine an individual’s health status.^21^ Sleep and PA are a pair of physical behaviors that have shown a synergistic and bidirectional relationship through behavioral and physiological pathways.^22,23^ A recent joint analysis found that <7 or >9 hours of device-measured sleep, combined with taking fewer daily steps, was adversely associated with metabolic health markers, including BMI, blood lipids, hypertension, and diabetes.^24^ However, no study, to date, has examined the joint association of device-measured sleep regularity and daily steps with cardiometabolic health.

The aim of our study was to examine the associations of different combinations (mutually exclusive groups) of device-measured sleep characteristics (regularity and duration) and daily step count with cardiometabolic health markers. We utilized the most extensive combined resource of thigh-worn accelerometry data currently available, the Prospective PA, Sitting, and Sleep Consortium, comprising 6 cohorts across 5 counties. Prospective PA, Sitting, and Sleep is a research platform that integrates both existing and future observational studies using wearable devices to analyze movement behaviors.^25^

## Methods

### Sample

Participants in the current study were drawn from the pooled Prospective PA, Sitting, and Sleep data resource^26,27^ consisting of 6 cohort studies: the ALSWH (Australian Longitudinal Study on Women’s Health, Australia; n=985),^28,29^ the BCS70 (1970 British Birth Cohort Study, United Kingdom; n=5250),^30^ the DPhacto (Danish Physical Activity Cohort With Objective Measurements, Denmark; n=834),^31^ the FIREA (Finnish Retirement and Aging Study, Finland; n=254),^32^ the NES (Nijmegen Exercise Study, the Netherlands; n=537),^33^ and TMS (The Maastricht Study, the Netherlands; n=7514).^34^ Ethical approval and informed consent were obtained at the cohort level, including permission for subsequent data analysis. A brief description of each study is presented in Table S1, with in-depth information available elsewhere.^25,29,31,32,35–37^ Following completion of appropriate data transfer agreements, data pooling, including harmonization of covariates and outcomes, and cleaning and processing of raw accelerometer data, was conducted at The University of Sydney. Our sample included participants with at least 3 days of valid accelerometer data (≥20 h of wear time and ≥3 h of sleep) and excluded participants with missing covariate or outcome data. The data that support the findings of this study are available from the Prospective PA, Sitting, and Sleep Consortium upon reasonable request, in accordance with cohort-specific regulations.

### PA and Sleep Measurements

Raw signal data on movement behaviors were obtained for 24 hours a day over 7 days using triaxial accelerometers worn on the anterior part of the thigh. Three different brands of accelerometers were used: ActivPAL (ALSWH, BCS70, NES, and TMS), Axivity (FIREA), and ActiGraph (Danish PA Cohort).^26,27^ There is a high accuracy and consistency in PA estimates across the different accelerometer brands.^38^ Although the ActiGraph is commonly worn on the hip, previous studies have shown the validity of thigh-worn ActiGraph under free-living conditions, where daily step estimates were highly correlated with Axivity and ActivPAL monitors worn on the thigh.^38^ We used the previously validated ActiPASS, version 1.34,^39^ software, to detect nonwear periods^40^ and process and harmonize raw accelerometer data.^41^ PA was identified using a decision tree–based deterministic algorithm,^42^ which has shown an accuracy over 90% for walking and running detection.^26^ Signal SD and tilt angle were used to identify walking activities and the signal frequency domain to estimate the number of steps.^42,43^ Step counts were categorized into low (<8475 steps/d), medium (8475–11 553 steps/d), and high (>11 553 steps/d) stepping groups based on the tertiles of the total daily step sample.

We calculated sleep duration using an algorithm validated against polysomnography for thigh placement, which detected sleep onset, offset, and awakenings to identify total sleep time.^44^ We categorized sleep duration into short (<7 h), adequate (7–8 h), and long (>8 h) based on modifications to the National Sleep Foundation sleep duration guidelines (self-report–based),^45^ considering our device-based measurements and sample distribution.^20,46^ Sleep regularity was assessed using the sleep regularity index (SRI).^47^ This index represents the percentage probability of an individual maintaining the same sleep/wake state at any 2 time points on adjacent days, with a scale from 0 (completely random) to 100 (perfectly regular).^47^ Based on SRI tertiles of our sample, we classified participants into low (<75.9), medium (75.9–84.5), and high (>84.5) sleep regularities, respectively.

### Cardiometabolic Outcomes

During home or clinic-based visits, researchers or trained health personnel from each cohort measured participants’ waist circumference (cm), height, and weight, which were used to calculate BMI (kg/m^2^; Table S2). Cardiometabolic blood biomarkers, including HDL cholesterol (mmol/L), total cholesterol(mmol/L), triglycerides (mmol/L), and HbA1c (mmol/mol), were measured from the blood samples provided by all participants, apart from the Danish PA Cohort where blood biomarkers were unavailable. Full assessment procedures and assay coefficients of variation are detailed in Table S3. We calculated standardized values for normalized cardiometabolic markers, using *Z* scores derived from the composite sample distribution.^48^ A composite score for cardiometabolic health was determined by taking the mean of the 6 normally distributed standardized scores, with a higher score indicating poorer cardiometabolic health.^26^ We inverted HDL cholesterol values as higher levels of HDL cholesterol are associated with protection against CVD.^49^

### Covariates

We selected covariates based on data availability and previous literature examining associations of sleep and PA with cardiometabolic risk markers.^8,24,26,27^ All cohorts provided information on participant age (years), sex (male/female), smoking status (nonsmoker/current smoker), alcohol consumption (tertiles based on weekly consumption), self-rated health (5-point Likert scale), self-reported medication use (blood pressure, glucose, and lipid-lowering) and self-reported history of CVD. A subset of cohorts provided additional information on education (n=4 cohorts; none or lower than high school, high school qualifications, further education qualifications, and university degrees and higher), occupational class (n=5; not working, low, intermediate, and high occupational class), diet (n=3; low, low-moderate, moderate-high, high fruit, and vegetable consumption), and mobility limitations (n=4, questionnaire scores ranging from 0 to 100). Full details of data harmonization procedures are provided elsewhere.^26,27^

### Statistical Analyses

We conducted a 1-stage individual participant data meta-analysis, in which raw individual participant data from multiple cohorts were harmonized into a single data set.^50^ The joint association between sleep and daily steps with risks of cardiometabolic outcomes was examined using generalized linear models.^48^ The outcome variables, composite score for cardiometabolic health and individual biomarkers, were treated as continuous. Generalized linear model coefficients represent the mean differences between the reference category and each of the other joint sleep and step count groups.^48^ For the sleep regularity-step combination, we categorized participants into mutually exclusive 9 combinations of sleep regularity (low, SRI <75.9; medium, 75.9≤SRI ≤84.5; and high, SRI >84.5 based on regularity tertiles) and step counts (low, <8475 steps/d; medium, 8475–11 553 steps/d; and high >11 553 steps/d). For the sleep duration-step combination, we similarly categorized participants into 9 combinations of sleep duration (short, <7 h; adequate, 7–8 h; and long, >8 h) and step counts (3 levels as above). In each analysis, we set the reference groups as the theoretically healthiest combination of sleep and daily steps, specifically, high sleep regularity and high step count, and adequate sleep duration (accounting for its U-shaped association with cardiometabolic health) and high step count. Models were adjusted for covariates available in all cohorts, including sex, age, smoking, alcohol, self-rated health status, medication use, prevalent CVD, and cohort. We also mutually adjusted for sleep duration and sleep regularity, that is, models examining sleep regularity were adjusted for sleep duration, and models examining sleep duration were adjusted for sleep regularity. Using data available only in the ALSWH, BCS70, DPhacto, and TMS cohorts, sensitivity analyses additionally adjusted separately for socioeconomic status (education), occupational class, diet (fruit and vegetable consumption), and mobility limitations. We also conducted a sensitivity analysis excluding participants with prevalent CVD or medication use. To assess the influence of missing accelerometry data on sleep regularity estimates in relation to social jetlag, we conducted a sensitivity analysis excluding participants with <7 days of valid accelerometer wear time (ensuring the inclusion of both weekdays and weekends). Additional sensitivity analyses were performed using the National Sleep Foundation age-specific sleep duration recommendation categorization (short <7 h, adequate 7–9 h, and long >9 h for adults aged 18–64 years; short <7 h, adequate 7–8 h, and long >8 h for adults aged ≥65 years) and using multiple imputation (chained equations with predictive mean matching method) to address missing covariate and outcome data. While compositional data analysis is a widely used method for examining relative proportions of time spent in different behaviors, it is not suited to our research question, which involved creating mutually exclusive participant groups and exploring sleep dimensions beyond total sleep time. All analyses were conducted using R statistical software (version 4.2.3) with the rms package.

## Results

### Sample

Our analytic sample size varied by outcome, ranging from 8892 for the composite cardiometabolic score to 11 903 for BMI (Figure S1). The maximum sample of 11 903 participants had a mean (SD) age of 54.7 (9.5) years, of which 54.9% were female. The average wear duration among participants with valid accelerometer data was 6.4 days. The sample showed a high level of activity with a median daily step count of 9950 steps/d and limited heterogeneity in sleep duration, with a median of 7 h/d and an SRI of 80.4. Overall, 56.3% participants were from the TMS cohort, 31.1% from BCS (British Birth Cohort Study), 7.6% from ALSWH, 2.2% from DPhacto, 1.7% from FIREA, and 1.0% from NES. Among all cohorts, NES was the most active, with a median daily step count of 12 076 steps/d, whereas TMS recorded the lowest median daily step count of 9620 steps/d. Detailed participant characteristics and cardiometabolic health markers, categorized by their total daily steps or sleep regularity, are presented in the Table and Table S4, respectively.

**Table.**
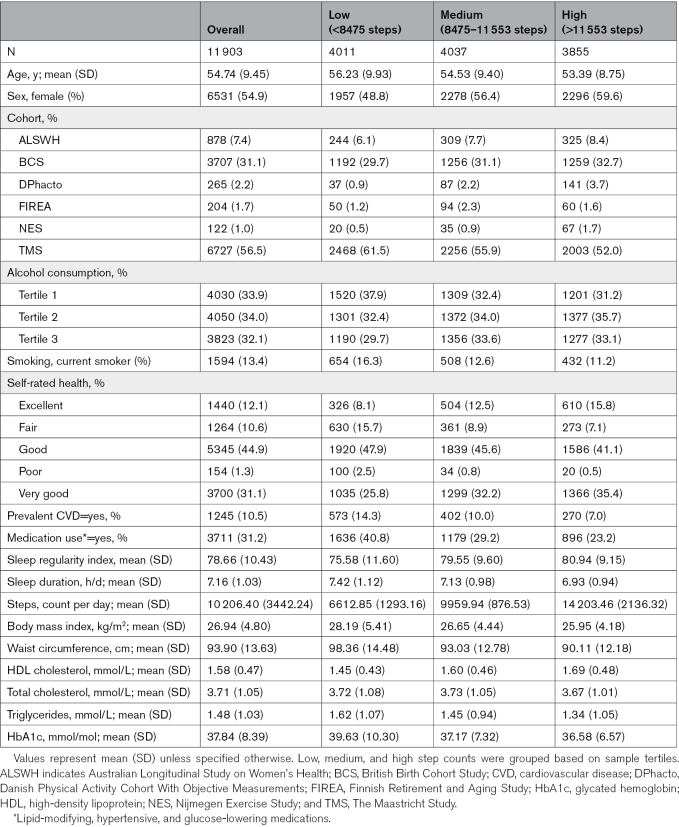
Participants’ Characteristics by Total Daily Steps (N=11 903)

### Joint Associations of Sleep and Steps With a Composite Cardiometabolic Health Score

**Figure 1.**
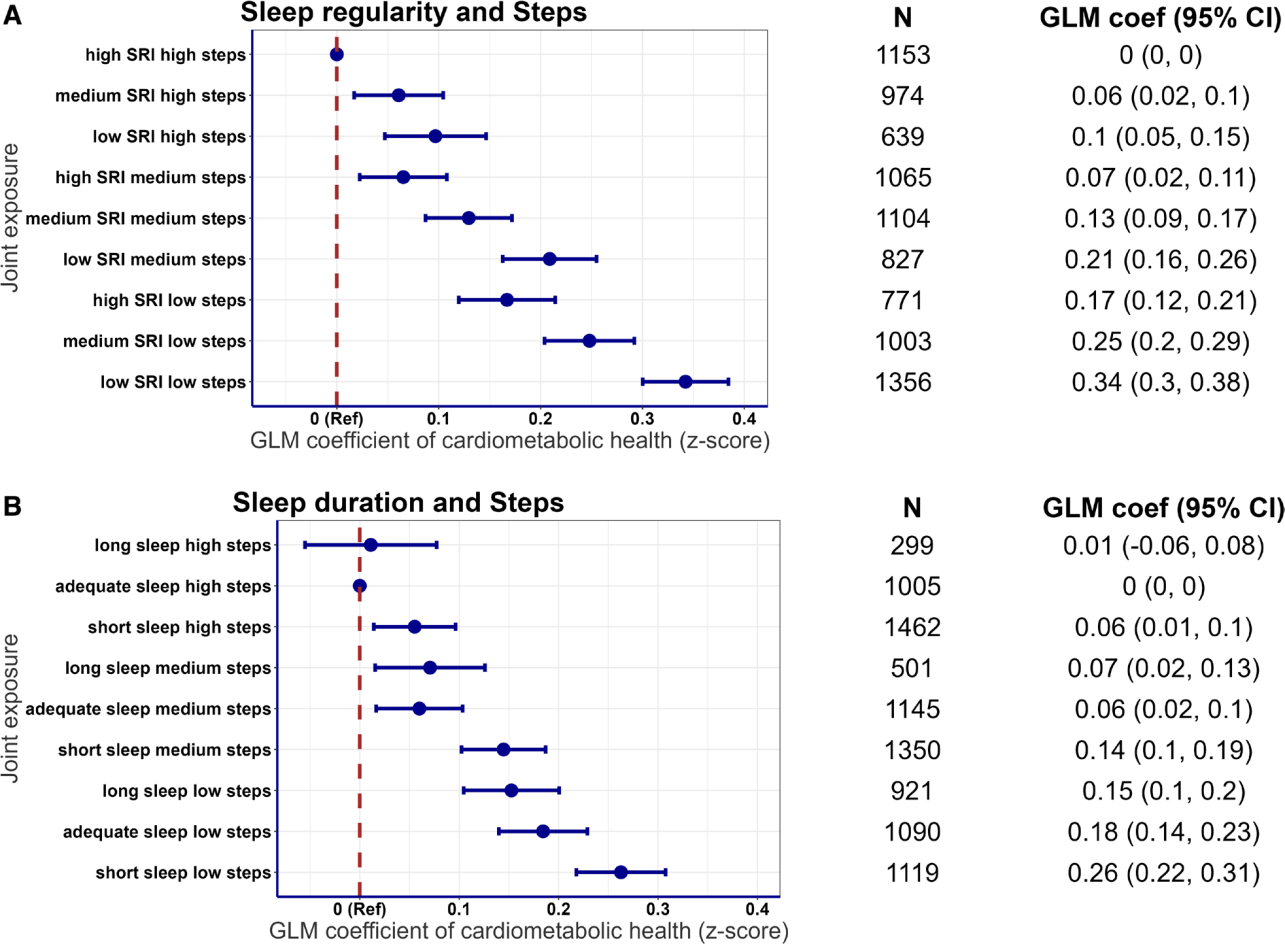
**Joint association of sleep regularity, sleep duration, and total daily steps with composite cardiometabolic health score (composite *Z* score: standardized mean score across cardiometabolic biomarkers).** Joint associations of (**A**) sleep regularity and total daily steps, and (**B**) sleep duration and total daily steps with composite cardiometabolic health (*Z* score, derived from the overall sample distribution; higher scores indicate poorer cardiometabolic health). Reference groups: (**A**) high sleep regularity index (SRI) and high total daily steps and (**B**) adequate sleep duration and high total daily steps. Sleep duration was categorized into short (<7 h/d), adequate (7–8 h/d), and long (>8 h/d); sleep regularity (low, SRI <75.9; medium, 75.9≤SRI ≤84.5; and high, SRI >84.5); and total daily steps (low, <8475 steps; medium, 8475–11 553 steps; and high, >11 553 steps) were categorized into tertiles. For instance, the joint exposure group, high SRI high steps, includes participants with a high SRI (SRI >84.5) and a high daily step count (>11 553 steps/d), while the joint exposure group, adequate sleep high steps, includes those with adequate sleep duration (7–8 h/d) and a high daily step count. Both models were adjusted for age, sex, cohort, smoking, alcohol consumption, education, self-rated health, medication use, prevalent cardiovascular disease, mobility limitations, and mutually adjusted for sleep duration and sleep regularity. N=8892. Generalized linear model (GLM) coefficients represent the mean differences of *Z* scores between the reference group and each of the other joint sleep and step groups.

Sleep regularity and steps, and sleep duration and steps showed clear and graded associations with overall cardiometabolic health (Figure 1). We observed that less regular sleep combined with lower total daily step count was associated with a more adverse cardiometabolic health score (ie, *Z* score >0; Figure 1A). For example, participants with lower SRI (<75.9) and lower daily step count (<8475 steps/d) had a higher *Z* score of 0.34 (95% CI, 0.3–0.38) compared with the reference group with high sleep regularity (SRI >84.5) and high daily steps (>11 553 steps/d). Both shorter (<7 h) and longer (>8 h) sleep durations combined with lower total daily step count were associated with more adverse cardiometabolic health profiles (Figure 1B). These results remained robust in all sensitivity analyses, including additional adjustment for education; N=8821 (Figure S2), occupation; N=7981 (Figure S3), fruit and vegetable consumption; N=7102 (Figure S4), mobility limitations; N=8843 (Figure S5); and when using multiple imputation for missing data; N=14 885 (Figure S6). The overall pattern of the joint association remained consistent after excluding individuals with prevalent CVD or medication use (N=5302; Figure S7); excluding those with <7 days of valid accelerometry wear time (N=6705; Figure S8); or recategorizing participants based on age-specific self-reported sleep duration recommendations (N=8892; Figure S9). Wider CIs were observed for the long sleep duration group likely due to smaller sample size and reduced statistical power.

### Joint Associations of Sleep Regularity and Steps With Individual Cardiometabolic Health Markers

**Figure 2.**
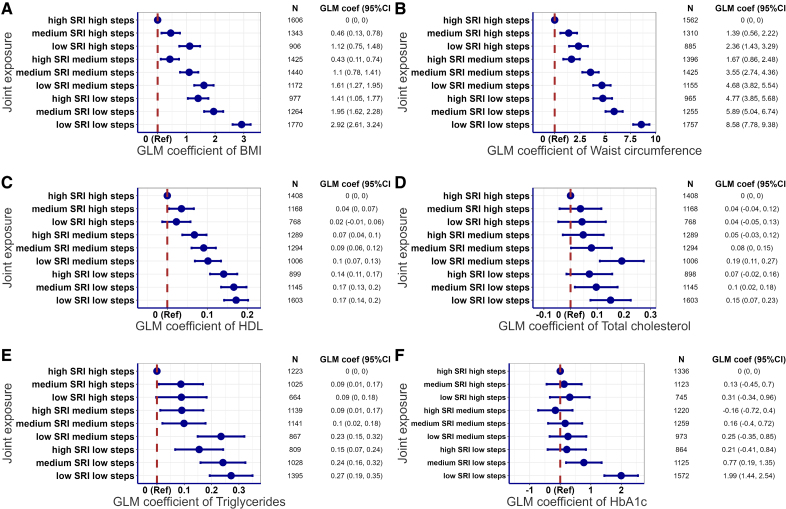
**Joint association of sleep regularity and total daily steps with body mass index (BMI), waist circumference, HDL (high-density lipoprotein), total cholesterol, triglycerides, and HbA1c (glycated hemoglobin).** Joint associations of sleep regularity and total daily steps with (**A**) BMI, (**B**) waist circumference, (**C**) HDL, (**D**) total cholesterol, (**E**) triglycerides, and (**F**) HbA1c. Reference group set to high sleep regularity index (SRI) and high total daily steps. Sleep regularity (low, SRI <75.9; medium, 75.9≤SRI ≤84.5; high; and SRI >84.5) and total daily steps (low, <8475 steps; medium, 8475–11 553 steps; and high, >11 553 steps) were categorized into tertiles. For instance, the joint exposure group, high SRI high steps, includes participants with a high SRI (SRI >84.5) and a high daily step count (>11 553 steps/d). Adjusted for age, sex, cohort, smoking, alcohol consumption, self-rated health, medication use, prevalent cardiovascular disease, and sleep duration. N=11 903 (BMI, kg/m^2^), 11 710 (waist circumference, cm), 10 580 (HDL, mmol/L), 10 579 (total cholesterol, mmol/L), 9291 (triglycerides, mmol/L), and 10 217 (HbA1c, mmol/mol). Generalized linear model (GLM) coefficients represent the mean differences between the reference group and each of the other joint sleep regularity and step groups.

Sleep regularity and daily steps showed a joint gradient, continuous decreasing trend, with some cardiometabolic biomarkers, including BMI, waist circumference, and HDL (Figure 2; Figures S10 through S14). Participants with less regular sleep and fewer daily steps had higher BMI (2.92 [2.61–3.24] kg/m^2^), waist circumference (8.58 [7.78–9.38] cm), total cholesterol (0.15 [0.07–0.23] mmol/L), and lower HDL (0.17 [0.14–0.2] mmol/L) compared with the reference group with high regular sleep and high daily steps (Figure 2A through 2D). We observed a clear joint gradient between sleep regularity and daily steps for BMI and waist circumference with a few deviations, such as the high regularity and medium daily steps group (Figure 2A and 2B). Lower step counts and, in most cases, more irregular sleep were associated with higher BMI and waist circumference. Sleep regularity and steps showed a near-linear joint gradient with HDL cholesterol (Figure 2C). There was less evidence of joint association between the 2 exposures and total cholesterol, triglycerides, or HbA1c. The detrimental association with triglycerides and HbA1c seemed to be primarily driven by a lower number of steps, as the joint gradient only appeared in the lower steps groups (Figure 2E and 2F). In contrast, the association with total cholesterol was more strongly influenced by less regular sleep patterns, specifically lower SRI scores (Figure 2D). Excluding participants with prevalent CVD and medication use or <7 days of valid accelerometer wear time had minimal influence on the overall pattern of the findings (Figures S15 and S16).

### Joint Associations of Sleep Duration and Steps With Individual Cardiometabolic Health Markers

**Figure 3.**
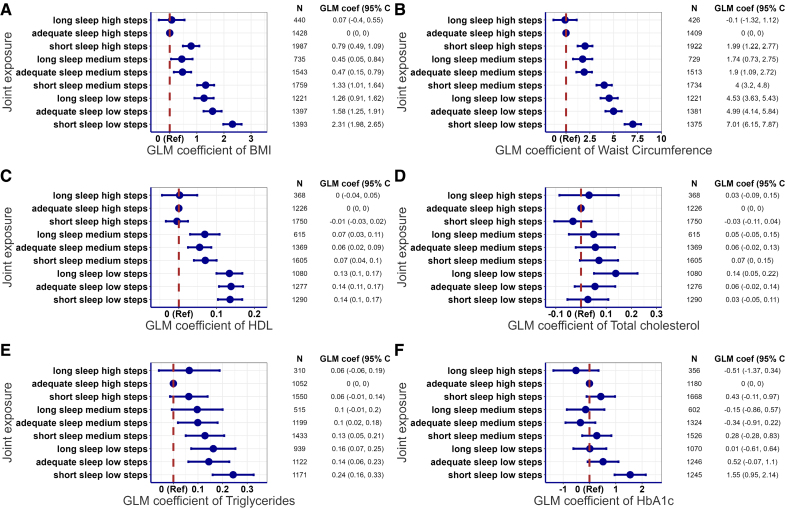
**Joint association of sleep duration and total daily steps with body mass index (BMI), waist circumference, HDL (high-density lipoprotein), total cholesterol, triglycerides, and HbA1c (glycated hemoglobin).** Joint associations of sleep duration and total daily steps with (**A**) BMI, (**B**) waist circumference, (**C**) HDL, (**D**) total cholesterol, (**E**) triglycerides, and (**F**) HbA1c. Reference group set to adequate sleep duration and high total daily steps. Sleep duration was categorized into short (<7 h/d), adequate (7–8 h/d), and long (>8 h/d), and total daily steps (low, <8475 steps; medium, 8475–11 553 steps; and high, >11 553 steps) were categorized into tertiles. For instance, the joint exposure group, adequate sleep high steps, includes participants with adequate sleep duration (7–8 h/d) and a high daily step count (>11 553 steps/d). Adjusted for age, sex, cohort, smoking, alcohol consumption, self-rated health, medication use, prevalent cardiovascular disease, and sleep regularity. N=11 903 (BMI, kg/m^2^), 11 710 (waist circumference, cm), 10 580 (HDL, mmol/L), 10 579 (total cholesterol, mmol/L), 9291 (triglycerides, mmol/L), and 10 217 (HbA1c, mmol/mol). Generalized linear model (GLM) coefficients represent the mean differences between the reference group and each of the other joint sleep duration and step groups.

Sleep duration and daily steps showed joint associations with cardiometabolic biomarkers, including BMI, waist circumference, HDL, and triglycerides (Figure 3; Figures S17 through S21). We observed joint gradients between sleep duration and daily step count with BMI and waist circumference (Figure 3A and 3B). Participants with shorter sleep duration and lower daily steps had a higher BMI (2.31 [1.98–2.65] kg/m^2^) and waist circumference (7.01 [6.15–7.87] cm). The joint association of the 2 exposures was less evident with HDL cholesterol. Daily step count showed significantly more pronounced direct associations with HDL cholesterol compared with sleep duration, with a lower number of daily steps being unfavorable (Figure 3C). We observed a nonsignificant joint gradient for triglycerides, with indications suggesting that both short and long sleep, and a low number of steps increase the risk of elevated triglycerides (Figure 3E). No joint association was found between sleep duration and total daily steps with total cholesterol and HbA1C (Figure 3D and 3F). Overall joint pattern did not change substantially after excluding participants with prevalent CVD and medication use (Figure S22) or <7 days of valid accelerometer wear time (Figure S23). The associations remained largely unchanged when participants were recategorized according to age-specific recommendations for sleep duration (Figure S24).

## Discussion

To our knowledge, this is the first individual participant data meta-analysis to explore the joint associations of objectively measured sleep patterns and daily step count with cardiometabolic health. Using a unique pooled harmonized resource, we found that sleep (regularity and duration) and daily step count were jointly associated with composite and individual cardiometabolic health markers after adjusting for potential confounders. Any deviation from the optimal pattern in either sleep (high sleep regularity and adequate sleep duration) or step counts (high daily step count) was adversely associated with the composite cardiometabolic health score. For instance, participants with low regularity or short duration of sleep and low number of total daily steps had significantly higher (worse) composite cardiometabolic health risks compared with those with high sleep regularity or adequate sleep duration and high step counts. While we observed similar joint associations across various individual biomarkers with irregular, inadequate sleep, and a low number of daily steps, in some instances, there were indications of a slightly different shape or nature of the associations.

### Comparison to Existing Evidence and Hypothesized Mechanisms

Our study offers novel insights into the joint associations of sleep regularity and step count with cardiometabolic health. These associations were more pronounced for BMI, waist circumference, and HDL cholesterol than for total cholesterol, triglycerides, and HbA1c. Low sleep regularity (SRI <75.9) combined with low total daily step count (≤8487 steps/d) was associated with higher BMI, waist circumference, total cholesterol, triglyceride, and lower HDL cholesterol. Irregular sleep timing was independently associated with adverse cardiometabolic health markers, including higher BMI^51^ and waist circumference,^52^ lower HDL cholesterol,^15^ and outcome events such as obesity, hypertension, and CVD.^53,54^ Studies have also shown that lower volumes of daily steps were associated with higher risk for obesity, diabetes, and cardiovascular events.^55–57^ Step count is a commonly used proxy for an individual’s overall PA. Lower levels of PA have been directly linked to insulin sensitivity through mechanisms such as decreased glucose uptake by muscles and altered lipid metabolism, leading to metabolic dysfunction and elevating the risk of morbidity and mortality related to type 2 diabetes.^58^ Irregular sleep disrupts the body’s natural circadian rhythms, which have been linked to metabolic dysregulation that may contribute to metabolic disorders, such as obesity and type 2 diabetes through alterations in insulin sensitivity, impaired glucose tolerance, and increased inflammation.^59^ The combination of irregular sleep patterns and a low volume of daily steps could potentially amplify the negative effects on cardiometabolic health relative to each factor on its own. Future studies should examine the prospective joint association of sleep regularity and stepping to understand the temporality between these combined exposures and cardiometabolic health outcomes.

Using accelerometry and multicohort meta-analysis, our study sheds light on areas with limited evidence, the joint associations between sleep duration and step counts with cardiometabolic health. In our study, a combination of short sleep duration (<7 h/d) with low step counts was associated with higher BMI, waist circumference, triglycerides, and lower HDL cholesterol compared with those getting adequate sleep (7–8 h/d) and taking more steps (over ≈11 000 steps/d). This association was particularly strong for BMI and waist circumference, which aligns with existing literature. For example, a systematic review reported that short sleep duration (<6 h/d) was associated with increased incidence of obesity, and both short (<6 h/d) and long (>8 h/d) sleep duration were associated with increased risk of incident type 2 diabetes.^17^ Sleep duration and daily step count have been found to be separately associated with obesity and type 2 diabetes.^17,55,56,60^ However, these movement behaviors are not entirely independent of each other, as shorter PA duration is associated with inadequate sleep duration.^61^ Our study supports findings from a recent analysis in the 1970 British cohort study, which showed that short sleep durations combined with low step counts are associated with higher BMI and elevated rates of hypertension and diabetes.^24^ Other cohort studies suggested that higher volumes of PA or moderate-to-vigorous PA attenuated the deleterious association of inadequate sleep duration with all-cause and cause-specific mortality outcomes.^62,63^

### Differences Across Cardiometabolic Outcomes

We also observed joint associations for sleep duration and total daily step count with HDL cholesterol and triglycerides though these relationships were less pronounced compared with BMI and waist circumference. For some outcomes, for example, HDL cholesterol, the joint associations were primarily driven by step counts. Specifically, lower daily steps were associated with lower HDL cholesterol compared with those with adequate sleep duration and high step counts, regardless of sleep duration. This suggests that overall, PA volume, as quantified by step count, could play a more important role in regulating cholesterol levels than duration of sleep. Although a Mendelian randomization analysis did not find statistically significant causal associations between moderate-to-vigorous PA or sleep duration and lipid levels, potentially due to directional pleiotropy,^64^ their results indicated that moderate-to-vigorous PA may be more strongly associated with HDL cholesterol levels than sleep duration. While longitudinal research is needed, our study suggests that striving for higher daily step count is a promising strategy for mitigating the deleterious associations of both short and long sleep durations, as well as irregular sleep, on cardiometabolic health markers.

### Comparison Between Sleep Patterns

A statistically significant joint association was found between sleep regularity and daily step count with HDL cholesterol, as well as some joint association with total cholesterol. However, no such association was evident for sleep duration and daily step count with HDL and total cholesterol. This might imply that maintaining sleep regularity may enhance the cardiometabolic health benefits of PA more effectively than merely ensuring adequate sleep duration. A recent prospective analysis using device-measured SRI and sleep duration found that sleep regularity was associated more strongly with all-cause and cause-specific mortality risk than sleep duration.^65^ Other studies have also shown that compared with inadequate sleep, irregular sleep can have a stronger adverse effect on some health outcomes, such as cardiometabolic risk^14^ and quality of life in cancer populations.^66^ Another cross-sectional study has shown that irregular sleep was more strongly associated with metabolic syndrome than sleep duration, potentially explaining the observed disparities in some cardiometabolic health outcomes.^67^ This result supports the idea of placing more importance on sleep regularity, providing fresh insights into lifestyle modifications aimed at improving cardiometabolic health and lipid profiles, and implications for the development of behavior change interventions and public health guidelines.

### Strengths and Limitations

Key strengths of our study include the large-scale pooled analyses that included participants from multiple cohorts across various countries, enhancing the generalizability of our findings, and the use of device-based exposure measurement that avoids potential bias from social desirability and poor recall.^19^ Moreover, the accelerometry placement on the thigh provides highly accurate detection of various types of activities, particularly ambulatory activities, using novel classification methods with over 90% accuracy^26,42^ and 80% accuracy for sleep detection.^44^ Harmonizing PA and sleep data across multiple cohorts significantly strengthened the robustness of the observed joint associations.^50,68^ This study simultaneously assessed 2 crucial aspects of sleep (regularity and duration), providing comprehensive insights into sleep’s complex multidimensional nature and its joint associations with daily steps on cardiometabolic health.

Our study also has several limitations, including the cross-sectional design that precludes causal interpretation and the possibility of unmeasured or residual confounding. For instance, we did not adjust for diet in our main models due to a substantial reduction in sample size, and short-term seasonal/weather-related variations in stepping behaviors were not accounted for. Also, we did not adjust for adiposity markers or other cardiometabolic biomarkers in our models to prevent overadjustment due to multicollinearity and the potential role of these variables as mutual mediators in the association between stepping or sleep behaviors and other risk factors.^69^ The strong joint associations of sleep patterns and step counts with adiposity markers observed in this study could potentially reflect a bidirectional relationship, where higher BMI or waist circumference may lead to poor sleep and lower levels of daily PAs. The presence of missing data in our sample could introduce bias. The categorization of continuous sleep and stepping variables may have resulted in a loss of granularity and reduced statistical power. It is noteworthy that despite the large sample size, the relatively healthy status of the samples in this study may limit the generalizability of the findings to populations with lower activity or poorer health. We chose total daily step count as the primary stepping metric to be included in the joint exposure, given its ease of interpretation and accessibility to the general population. Step count is also commonly captured by consumer wearables and may hold potential as a basis for future digitally assisted health interventions. To enhance the generalizability and reliability of our findings, future research involving diverse ethnicities and populations with varying activity levels, additional stepping metrics (such as cadence-defined stepping intensity), or incorporating longitudinal data is warranted.

### Conclusions

Our study provides novel evidence on the joint associations between sleep patterns and daily step count with cardiometabolic health by using a pooled multicohort individual participant data meta-analysis and device-based measures. Irregular sleep patterns or inadequate sleep duration combined with lower total daily step count may be adversely associated with cardiometabolic health. More studies on similar contexts are required to provide guidance to future interventions and prospective studies.

## Article Information

### Acknowledgments

The data on which this research is based were drawn from 6 observational studies. The research included data from the ALSWH (Australian Longitudinal Study on Women’s Health) from the University of Newcastle, Australia, and The University of Queensland, Australia. The authors are grateful to the Australian Government Department of Health and Aged Care for funding and to the women who provided the survey data. The authors thank the following Prospective Physical Activity, Sitting, and Sleep Consortium collaborators for their contributions to the article: H. Savelberg (Department of Human Movement Sciences, School for Nutrition and Translational Research in Metabolism, Maastricht University, Maastricht, the Netherlands; B. de Galan and C. van de Kallen (Department of Internal Medicine, Maastricht University Medical Centre, Cardiovascular Research Institute Maastricht, Maastricht University, Maastricht, the Netherlands; and T. M. H. Eijsvogels (Department of Medical BioSciences, Radboud University Medical Center, Nijmegen, the Netherlands).

### Sources of Funding

This study was funded by the British Heart Foundation (grant SP/F/20/150002). The establishment of the Prospective Physical Activity, Sitting, and Sleep (ProPASS) Consortium was supported by an unrestricted 2018-20 grant from PAL Technologies (Glasgow, United Kingdom). Several aspects of the ProPASS Consortium methods used in this article were funded by a National Health and Medical Research Council Ideas Grant (APP1194510). The Charles Perkins Centre (The University of Sydney, Australia) and the National Research Centre for the Working Environment (Copenhagen, Denmark) cofunded the technical proof-of-concept study of the ProPASS Consortium that enabled the pooling of data from different brands of wearables. Dr Hamer is supported through the NIHR (National Institute for Health and Care Research) University College London Hospitals Biomedical Research Centre (grant NIHR203328). Dr Stamatakis is funded by a National Health and Medical Research Council Investigator Grant (APP1194510). Dr del Pozo Cruz is supported by the Government of Andalusia, Research Talent Recruitment Programme (grant EMERGIA 2020/00158). Dr Mielke is supported by a National Health and Medical Research Council Investigator Grant (APP2008702). Dr Mishra is supported by a National Health and Medical Research Council Principal Research Fellowship (grant APP1121844). Dr Bakker received funding from the European Union’s Horizon 2020 Research and Innovation Program under the Marie Skłodowska-Curie grant agreement 101064851. FIREA (Finnish Retirement and Aging Study) is supported by the Academy of Finland (grants 286294, 294154, 319246, and 332030), the Ministry of Education and Culture, the Juho Vainio Foundation, and the Finnish State Grants for Clinical Research. ActiPASS development was partly funded by FORTE, the Swedish Research Council for Health, Working Life and Welfare (grant 2021–01561). ALSWH (Australian Longitudinal Study on Women’s Health) is funded by the Australian Government Department of Health and Aged Care, and its substudy, from which accelerometry and clinical data were obtained, was funded by a National Health and Medical Research Council Project Grant (APP1129592). Dr Ahmadi is supported by the National Heart Foundation (grant APP107158). The funder had no specific role in any of the following study aspects: the design and conduct of the study; collection, management, analysis, and interpretation of the data; preparation, review, or approval of the article; and the decision to submit the article for publication.

### Disclosures

Dr Stamatakis is a paid consultant and holds equity in Complement Theory, Inc, a US-based startup whose products and services relate to physical activity. The other authors report no conflicts.

### Supplemental Material

Tables S1–S4

Figures S1–S24

## Supplementary Material
